# STAT3‐activated lncRNA XIST accelerates the inﬂammatory response and apoptosis of LPS‐induced acute lung injury

**DOI:** 10.1111/jcmm.16653

**Published:** 2021-06-11

**Authors:** Jun Li, Lei Xue, Yunfei Wu, Qiang Yang, Degang Liu, Changhui Yu, Jiangzhou Peng

**Affiliations:** ^1^ Department of Thoracic Surgery The Third Affiliated Hospital of Southern Medical University Guangzhou China; ^2^ Department of Thoracic Surgery The Affiliated Hospital of Southwest Medical University Luzhou China; ^3^ Department of Respiratory and Critical Care Medicine Chronic Airways Diseases Laboratory Nanfang Hospital Southern Medical University Guangzhou China

**Keywords:** acute lung injury, lipopolysaccharide, lncRNA, STAT3, XIST

## Abstract

Acute lung injury (ALI) is a severe lung respiratory failure characterized by high morbidity and mortality. Novel findings demonstrated the critical roles of long non‐coding RNA (lncRNA) in ALI. Here, we tried to investigate the roles and potential mechanism of lncRNA X‐inactive specific transcript (XIST) in ALI. Results illustrated that lncRNA XIST was up‐regulated in the lipopolysaccharide (LPS)‐induced ALI mice models and pulmonary endothelial cells. Biofunctional assays unveiled that knockdown of XIST repressed the inﬂammatory response and apoptosis in LPS‐induced endothelial cells. Mechanistically, XIST acted as the miR‐146a‐5p sponge to positively regulate STAT3. Moreover, STAT3 combined the promoter region of XIST to accelerate the transcription, constituting the positive feedback loop of XIST/miR‐146a‐5p/STAT3 in ALI. Collectively, these findings suggested that XIST knockdown attenuates the LPS‐induced ALI, providing a potential therapeutic target.

## 1 INTRODUCTION

Acute lung injury (ALI) is a devastating respiratory disorder and severe clinical condition, which is characterized by the injury of alveolar epithelial cells and capillary endothelial cells.^1^, ^2^ ALI and its heavy form acute respiratory distress syndrome (ARDS) are a result of various situations, including pneumonia, trauma, sepsis, acute pancreatitis and so on.^3^ In the ALI pathophysiological process, multiple factors are involved, including infiltration of inflammatory cells, pro‐inflammatory mediator production and alveolar epithelial cell apoptosis.^4^ Although significant advancements have been achieved, the annual mortality of ALI is still astonishing.^5^ Examining the underlying mechanisms of ALI may provide a new approach to develop individualized therapeutic strategies and to investigate potential combination therapies for the ALI. Given the importance of targeted therapy, the ascertainment of accurate therapeutic targets is considered to be very critical.

Long non‐coding RNAs (lncRNAs) are the majority of transcripts generated from human genome.^6^ The numerous transcripts without protein‐coding potential have been discovered to play critical roles in multiple pathophysiological processes, including ALI.^7^ For example, the knockdown of lncRNA MALAT1 exerts a protective role in the LPS‐induced ALI rat model and inhibited LPS‐induced inflammatory response in murine alveolar epithelial cells and murine alveolar macrophages cells through sponging miR‐146a.^8^ LncRNA H19 protects the LPS‐mediated MRC‐5 cell injury by inhibiting miR181, which in turn promotes the Runx2 expression to activate Notch and JNK pathways.^9^ Collectively, the growing body of literature suggests that lncRNAs may remarkably regulate the pathophysiological process of ALI.

Here, the present research found that lncRNA XIST and STAT3 were both up‐regulated in the LPS‐induced ALI model. Mechanistically, lncRNA XIST functioned as a miRNA sponge to absorb the expression of miRNA, thereby up‐regulating the STAT3 level. Of note, transcription factor STAT3 in turn binds with the promoter region of XIST to accelerate the XIST expression.

## 2 MATERIALS AND METHODS

### 2.1 Establishment of ALI model

The ALI mice model induced by LPS was established as previously reported.^10^ C57BL/6 mice were purchased from Shanghai Slack Laboratory Animal Company (Shanghai, China) and then randomly divided into ALI group (treated with 3 mg/kg LPS) and control group (1.5 mg/kg normal saline) through intra‐tracheal instillation. Bronchoalveolar lavage fluid (BALF) was collected and centrifuged at 4°C for 15 minutes using cooling centrifuge. This assay was approved by the Ethics Committee of The Third Affiliated Hospital of Southern Medical University. Then, human PMVECs were purchased from ScienCell Research Laboratories and cultured in Dulbecco's modified Eagle medium (DMEM) added with 10% FCS (foetal calf serum, HyClone). PMVECs (pulmonary microvascular endothelial cells) were administrated with lipopolysaccharide (LPS, 50 ng/mL, S1732‐25, Beyotime) to mimic the ALI cell model as previously reported.^11^

### 2.2 Transfection

The lentivirus packaging and transduction were performed by Shanghai Genechem Co., Ltd. The lentivirus‐mediated silencing vectors containing XIST shRNA were cloned into a pLKD‐CMV‐G&PR‐U6‐shRNA vector. After the transfection in PMVECs with 5 mg/mL polybrene, puromycin (2 mg/mL) was added to select the infected cells. miR‐146a‐5p mimic and inhibitor were purchased from GenePharma. The indicated shRNA sequences are listed in Supplementary Table S1.

### 2.3 RNA isolation and quantitative real‐time PCR

Total RNA was isolated from LPS‐induced PMVECs cells and tissues using TRIzol reagent (Invitrogen). Synthesis of first‐strand cDNA was performed with the instructions of PrimeScript RT reagent kit (Takara). Real‐time PCR was performed on the 7900 Real‐time PCR System using Fast SYBR green master mix (Life Technologies) and TaqMan microRNA assay kit (Thermo Fisher Scientific). The qRT‐PCR data were normalized using the endogenous GAPDH and U6 as internal controls for mRNA or miRNA. The relative gene expression of GAPDH and U6 was calculated using the comparative delta‐delta CT method (2^‐ΔΔCt^). Additional sequences are provided in Supplementary Table S1.

### 2.4 Western blot

Total protein was extracted from PMVECs using radioimmunoprecipitation assay (RIPA) analysis buffer. After the extraction, the protein was qualified using BCA protein assay kit (Beyotime). Equal amount (20 μg/lane) of protein isolation was loaded and separated on 10% sodium dodecyl sulphate polyacrylamide gel electrophoresis (SDS‐PAGE) and then transferred to polyvinylidene diﬂuoride membranes. Subsequently, members were blocked with skim milk (5%) in a phosphate‐buffered saline with Tween solution (50 mM Tris, 0.1% Tween‐20, 100 mM NaCl, PH 7.5) at room temperature and then incubated by indicated primary antibodies (anti‐STAT3, 1:1000, Abcam, ab76315) at 4 ℃ overnight. After the primary antibody, membranes were incubated with horseradish peroxidase (HRP)–conjugated secondary antibodies (Sigma‐Aldrich, Saint Louis, USA) and visualized by enhanced chemiluminescence (ECL) and detected with ImageJ software.

### 2.5 Flow cytometry apoptosis analysis

The apoptosis of PMVECs was analysed by flow cytometry as described previously. Briefly, cells were harvested and stained with fluorescein isothiocyanate (FITC)‐Annexin V and propidium iodide using the FITC Annexin V Apoptosis Detection Kit (BD Biosciences) according to the manufacturer's protocols. The relative ratio of early and later apoptotic cells was calculated as compared with control transfection for each experiment.

### 2.6 Enzyme‐linked immunosorbent assay (ELISA)

The functional activity of inflammatory cytokines was analysed by the enzyme‐linked immunosorbent assay (ELISA). The concentration of inflammatory cytokines (IL‐6, IL‐1β, TNF‐α) was detected using specific ELISA kits according to the manufacturer's protocol and expressed as pg per millilitre.

### 2.7 Fluorescence in situ hybridization (FISH)

The cy3‐labelled probe for XIST was constructed by Genepharma. RNA‐FISH was performed using fluorescent in situ hybridization kit (Genepharma) according to the manufacturer's protocol. After pre‐hybridization, slides were incubated with pre‐hybrid buffer with 0.1 μg biotin‐labelled probe at 33°C overnight and washed (37°C, 10 minutes). Then, slide was incubated with labelled streptavidin at room temperature and washed with PBS. The signals of locked nucleic acid miR‐146a‐5p probes were detected using a tyramide‐conjugated Alexa 488 fluorochrome TSA kit. Nuclei were counterstained with 4,6‐diamidino‐2‐phenylindole (DAPI). The image was obtained with a confocal microscope (Olympus).

### 2.8 Subcellular fractionation location

The cytoplasmic or nuclear fraction was isolated using PARIS Kit (Invitrogen) following the manufacturer's protocol.

### 2.9 Chromatin immunoprecipitation (ChIP)

ChIP‐PCR analysis was performed according to the Magna ChIP Chromatin Immunoprecipitation Kit according to the manual (Millipore). Briefly, chromatin fragments (200 to 1000 bp) were generated from cross‐linked status through sonicating. Anti‐STAT3 antibody (anti‐STAT3, 1:1000, Abcam, ab76315) was used to precipitate DNA‐protein complex. Normal immunoglobulin G (IgG) was used as a negative control. The precipitated DNA was quantified using qRT‐PCR.

### 2.10 Luciferase reporter assay

The XIST promoter reporter plasmid pGL3‐XIST and the 3′‐UTR (XIST and STAT3 mRNA) were generated by amplification and then subcloned into the luciferase reporter pGL3 vector (Promega). The wild type (WT) and mutant (MUT) were constructed and transfected into the 293T cells, as well as the miR‐146a‐5p mimics. Then, the Fireﬂy/Renilla luciferase activity was measured after 24 hours using DualGlo luciferase assay system (Promega). All experiments were performed in triplicate and repeated three times.

### 2.11 Data analysis

Statistical analyses were performed using GraphPad Prism (version 7.0; GraphPad Software) by *t* test or ANOVA with Dunnet's least significant difference post hoc tests. Data were displayed as mean ± standard error of the mean (SEM). A *P*‐value <.05 was considered as significant.

## 3 RESULTS

### 3.1 LncRNA XIST was up‐regulated in LPS‐induced ALI

**FIGURE 1:**
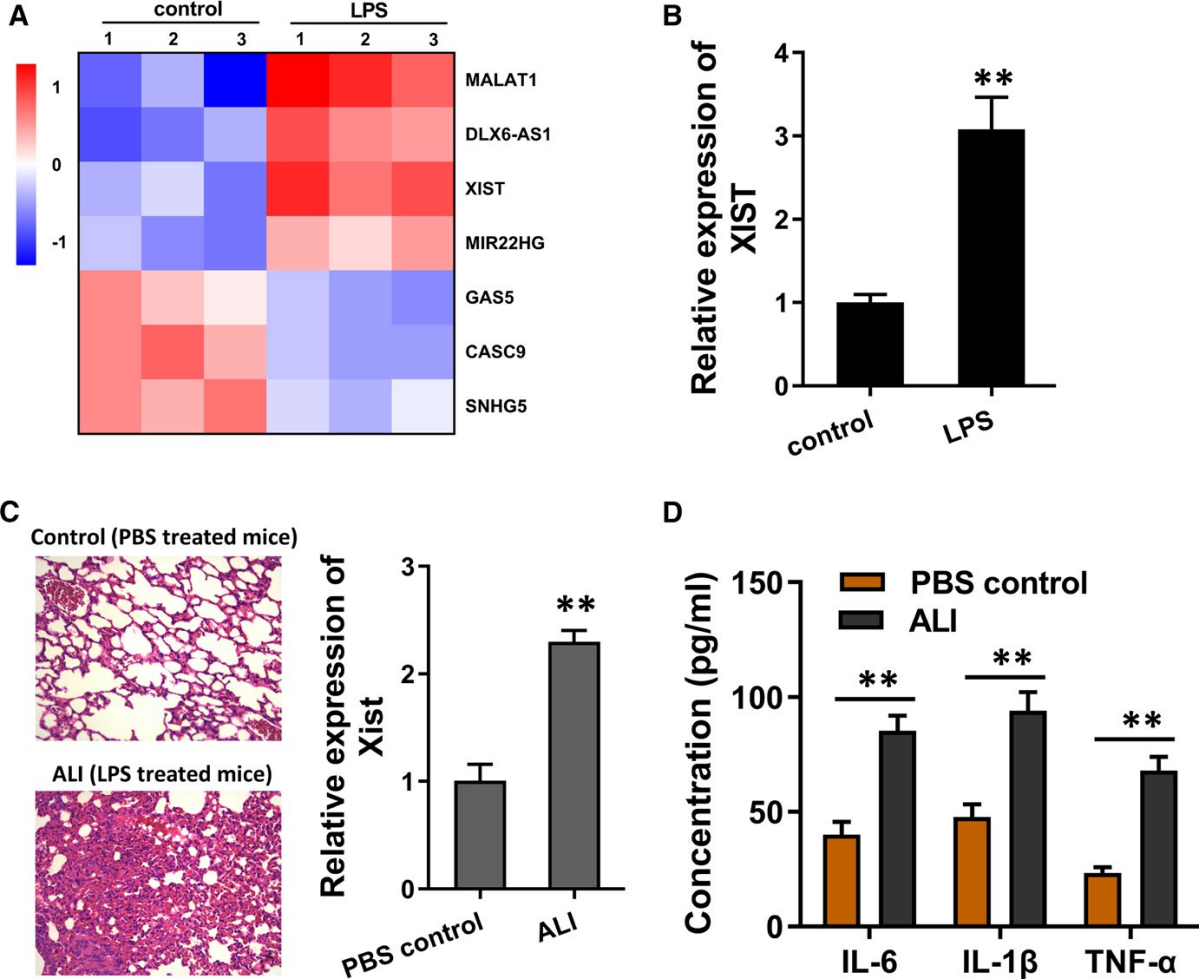
LncRNA XIST was up‐regulated in LPS‐induced ALI. A, Heatmap demonstrated the differently expressed multiple alternative candidate lncRNAs in LPS‐induced or control human PMVECs. B, RT‐PCR demonstrated the expression of lncRNA XIST in LPS‐induced PMVECs as compared to the control. C, The LPS‐induced mice models were constructed to mimic the ALI. LncRNA XIST was detected using RT‐PCR in LPS‐induced mice ALI models and controls. D, ELISA was performed in bronchoalveolar lavage fluid (BALF) to detect the enrichment of inflammatory cytokines (IL‐6, IL‐1β, TNF‐α). **P < .01

In order to investigate the potentially dysregulated lncRNAs in the ALI models, LPS‐induced PMVECs were constructed to mimic the pathophysiological process. Multiple alternative candidate lncRNAs were detected and displayed using heatmap (Figure 1A). Moreover, the expression of lncRNA XIST was found to be up‐regulated in LPS‐induced PMVECs as compared to the control cells (Figure 1B). Then, in the LPS‐induced mice ALI models, lncRNA XIST was also remarkably up‐regulated (Figure 1C). ELISA demonstrated that the enrichment of inflammatory cytokines (IL‐6, IL‐1β, TNF‐α) was significantly increased in the mice ALI models as compared to the control group (Figure 1D). In summary, these data suggested that lncRNA XIST was up‐regulated in LPS‐induced ALI.

### 3.2 Transcription factor STAT3 accelerated the transcriptional level of XIST in LPS‐induced PMVECs

**FIGURE 2:**
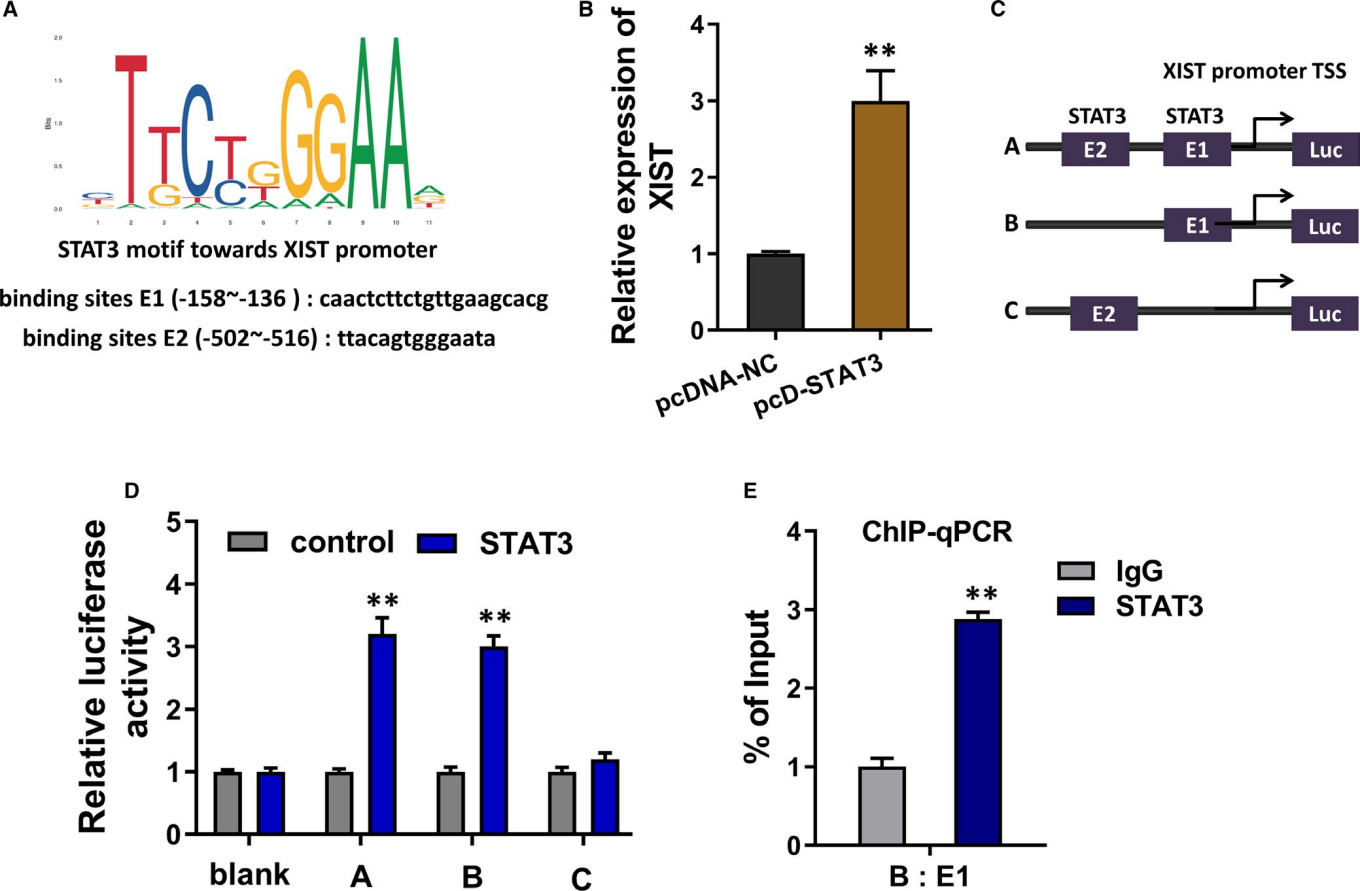
Transcription factor STAT3 accelerated the transcriptional level of XIST in LPS‐induced PMVECs. A, Online bioinformatics tools (RegRNA 2.0, http://regrna2.mbc.nctu.edu.tw/; GEPIA, http://jaspar.genereg.net/) demonstrated that transcription factor STAT3 shared several binding sites on the XIST promoter region. B, RT‐qPCR demonstrated the XIST expression in the LPS‐induced PMVECs transfected with STAT3 overexpression plasmids (pcD‐STAT3) or control (pcDNA‐NC). C, Luciferase reporter vectors were constructed, including the E1 and/or E2. D, Chromatin immunoprecipitation (ChIP)‐qPCR assays showed the enrichment of precipitated XIST promoter region fraction incorporated by anti‐STAT3 antibody. **P < .01

Previous research had found that STAT3 functioned as a critical regulator in the ALI; besides, STAT3 was a considerable substantial transcription factor.^12^, ^13^ In the present research, we found an interesting phenomenon that STAT3 shared several binding sites on the XIST promoter region (Figure 2A). In LPS‐induced PMVECs, the enforced STAT3 expression was constructed and the expression of XIST was up‐regulated in the STAT3 overexpression transfection (Figure 2B). Then, in the luciferase reporter assay, we constructed the luciferase reporter vector, concluding the E1 and/or E2 (Figure 2C). Luciferase reporter assay results demonstrated that the first region (E1) displayed the potential binding capacity towards transcription factor STAT3 (Figure 2D). Chromatin immunoprecipitation (ChIP)‐qPCR assays showed that STAT3 could significantly combine with the first region (E1) of XIST promoter region (Figure 2E). In summary, these data demonstrated that transcription factor STAT3 accelerated the transcriptional level of XIST in LPS‐induced PMVECs.

### 3.3 Knockdown of XIST repressed the inﬂammatory response and apoptosis of LPS‐induced PMVECs

**FIGURE 3:**
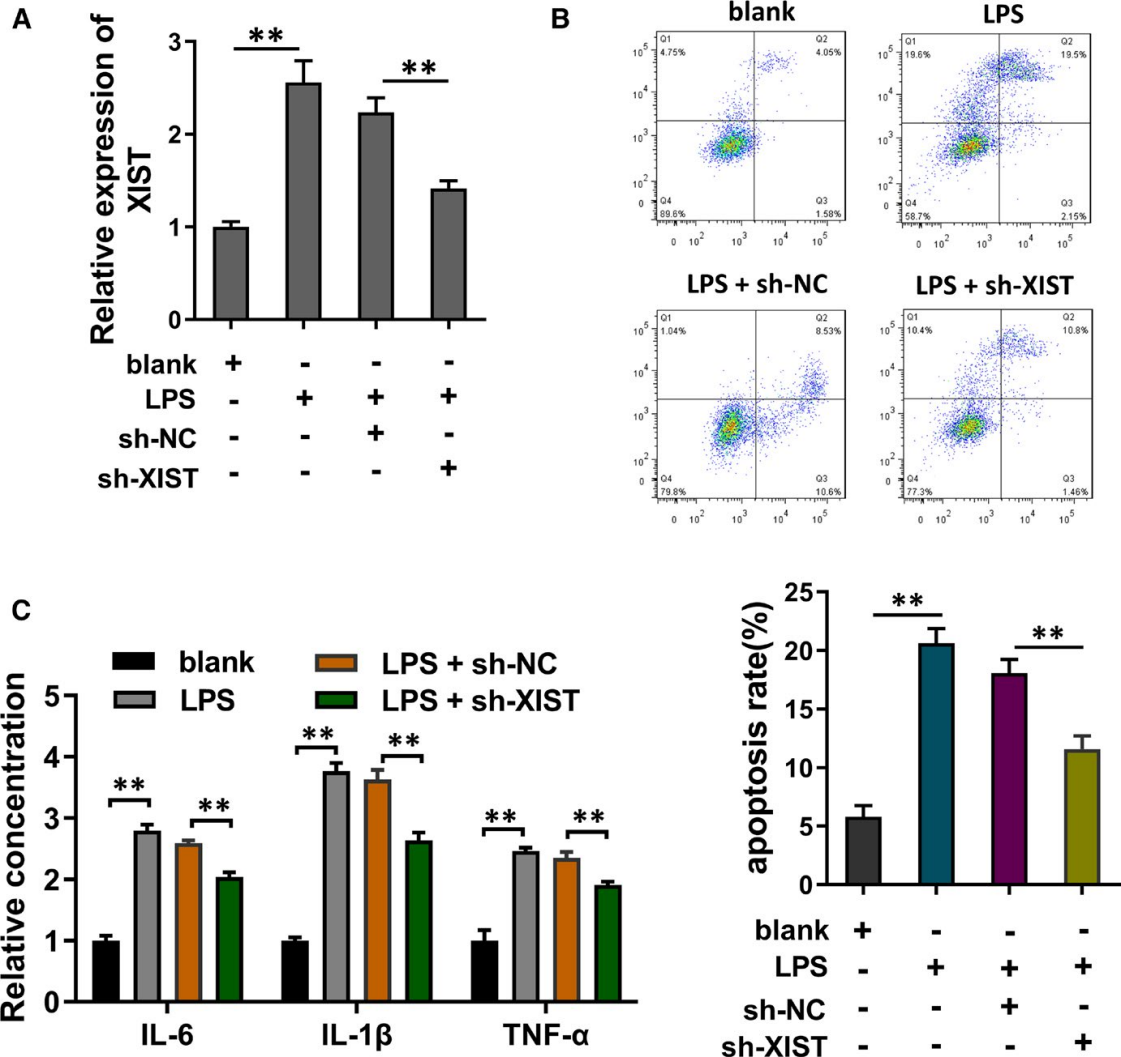
Knockdown of XIST repressed the inﬂammatory response and apoptosis of LPS‐induced PMVECs. A, The XIST knockdown (shRNA targeting XIST, sh‐XIST) as well as control (sh‐NC) were transfected in LPS‐induced PMVECs. XIST level was detected using RT‐PCR. B, Flow cytometry analysis showed the apoptosis in LPS treatment and XIST knockdown. C, ELISA demonstrated the enrichment of inflammatory cytokines (IL‐6, IL‐1β, TNF‐α) in the LPS‐induced PMVECs and XIST knockdown transfection. **P < .01

To investigate roles of XIST in ALI, the XIST knockdown (shRNA targeting XIST, sh‐XIST) was transfected in LPS‐induced PMVECs, which was detected using RT‐PCR (Figure 3A). Flow cytometry analysis found that LPS treatment caused the apoptosis increasing, while the XIST knockdown reduced the apoptosis (Figure 3B). ELISA demonstrated that the enrichment of inflammatory cytokines (IL‐6, IL‐1β, TNF‐α) was significantly increased in the LPS‐induced PMVECs, while reduced in the XIST knockdown transfection (Figure 3C). In conclusion, these data suggested that knockdown of XIST repressed the inﬂammatory response and apoptosis of LPS‐induced PMVECs.

### 3.4 XIST was targeted by miR‐146a‐5p at 3′‐UTR

**FIGURE 4:**
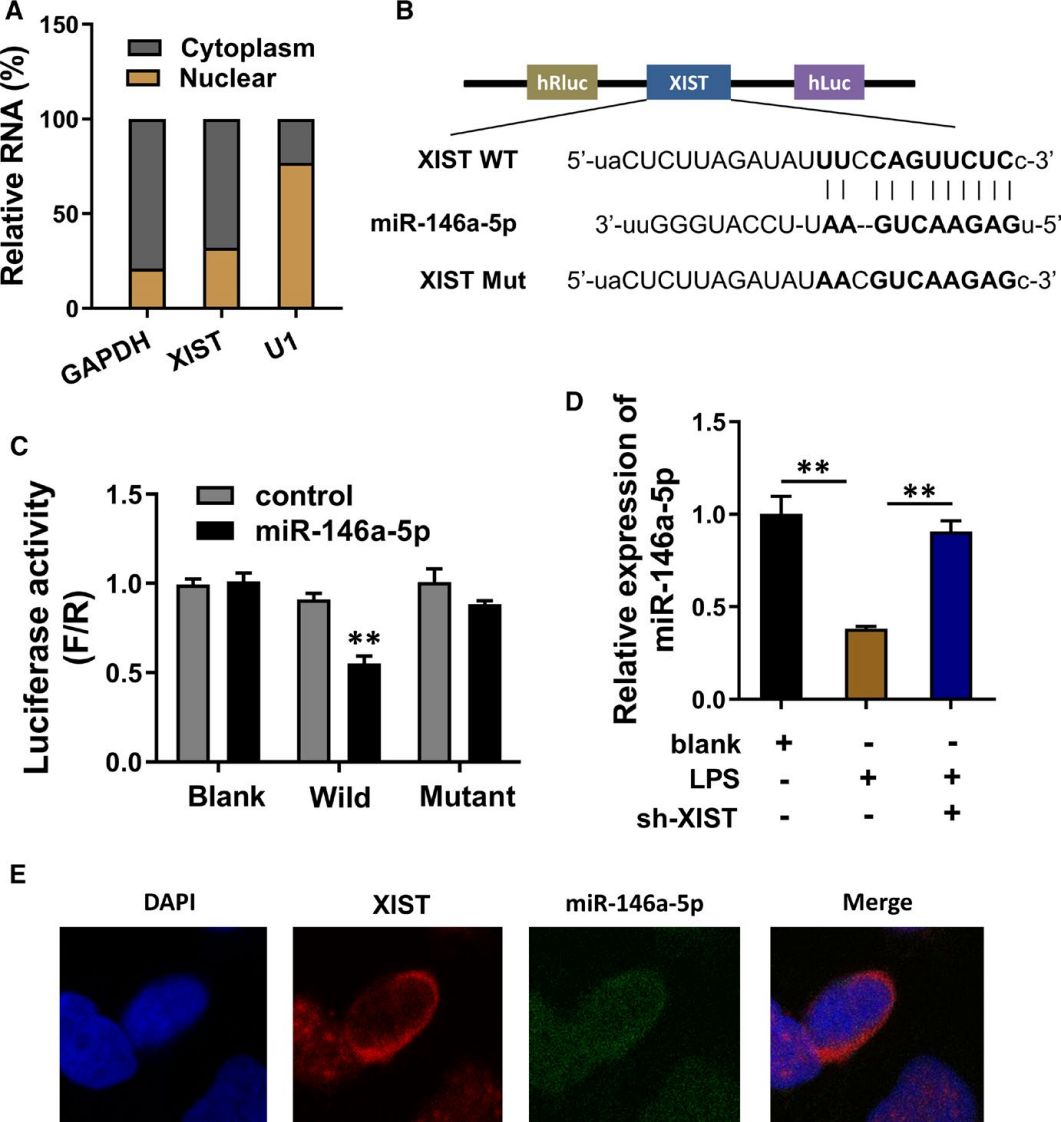
XIST was targeted by miR‐146a‐5p at 3′‐UTR. A, Subcellular fractionation location analysis was performed to detect the location of XIST in PMVECs. B, Bioinformatics prediction approach (EOCORI, http://starbase.sysu.edu.cn/) demonstrated the targeting sites within miR‐146a‐5p and XIST 3′‐UTR. C, Luciferase reporter assay was performed to identify the binding within miR‐146a‐5p and XIST wild type (WT), rather than mutant. D, Quantitative RT‐PCR was performed to detect the miR‐146a‐5p expression in control or LPS‐induced PMVECs. E, RNA fluorescence in situ hybridization (RNA‐FISH) illustrated the distribution and colocalization of XIST and miR‐146a‐5p in PMVECs. **P < .01

In the initial detection, we found that XIST was predominantly located in the cytoplasm of PMVECs (Figure 4A). Bioinformatics prediction approach (EOCORI, http://starbase.sysu.edu.cn/) demonstrated that miR‐146a‐5p could target the 3′‐UTR of XIST (Figure 4B), which was validated by the luciferase reporter assay (Figure 4C). Moreover, the expression of miR‐146a‐5p was detected using RT‐PCR. Data showed that miR‐146a‐5p was decreased in the LPS administration, which was recovered in the transfection of XIST shRNA (Figure 4D). RNA fluorescence in situ hybridization (RNA‐FISH) illustrated that XIST and miR‐146a‐5p were both predominantly distributed in cytoplasm of PMVECs (Figure 4E). In conclusion, these data suggested that XIST was targeted by miR‐146a‐5p at 3′‐UTR.

### 3.5 STAT3 functioned as the downstream target of XIST/miR‐146a‐5p axis

**FIGURE 5:**
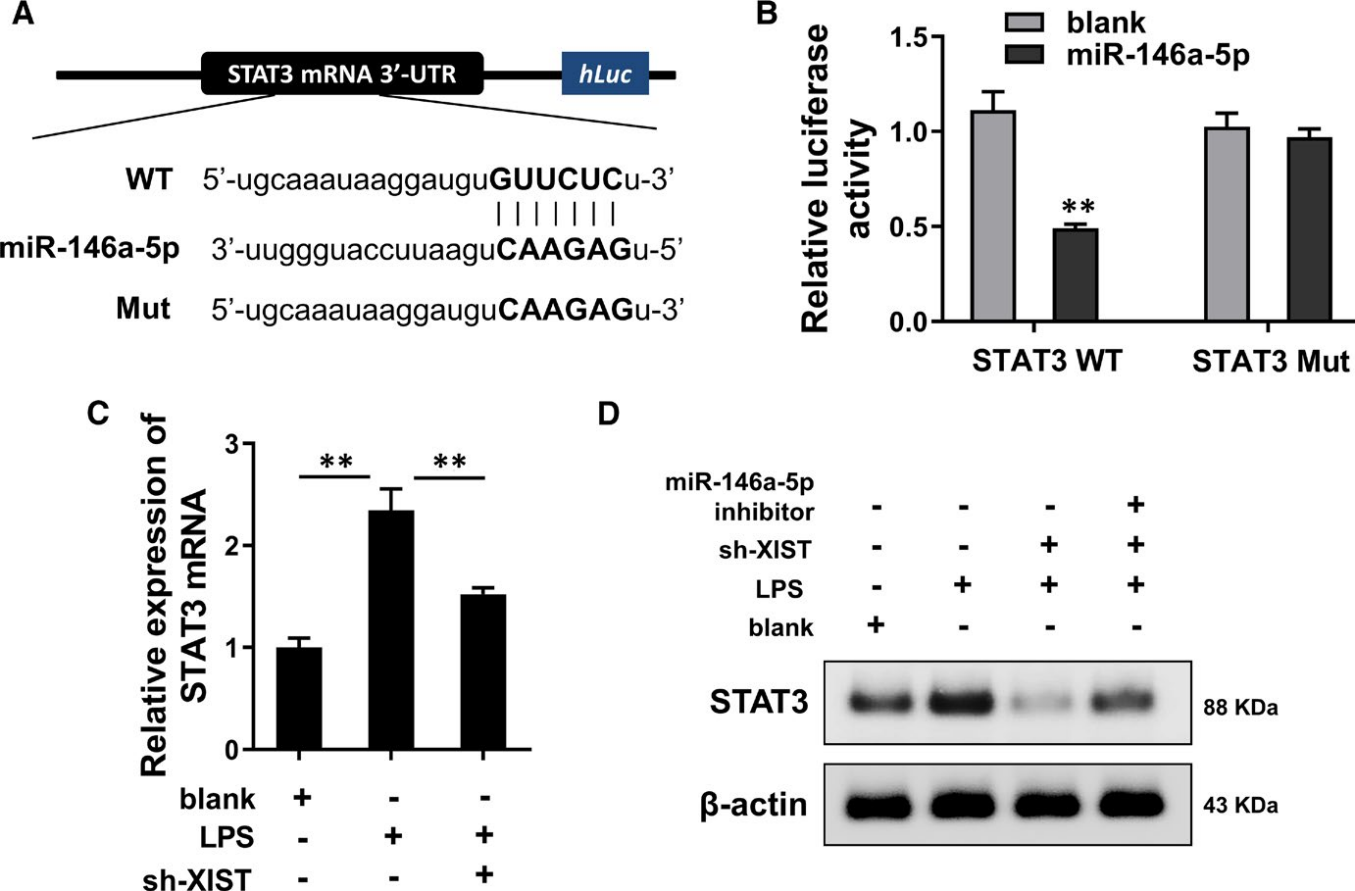
STAT3 functioned as the downstream target of XIST/miR‐146a‐5p axis. A, Bioinformatics prediction inspired the targeting of STAT3 wild type and miR‐146a‐5p. B, Luciferase reporter assay demonstrated the significant combining of miR‐146a‐5p mimics with the STAT3 mRNA 3′‐UTR. C, RT‐PCR assay demonstrated the STAT3 mRNA levels in the LPS treatment and XIST knockdown (sh‐XIST) transfection. D, Western blot analysis showed the STAT3 protein level in the LPS treatment or XIST knockdown (sh‐XIST) or co‐transfection of miR‐146a‐5p inhibitor with sh‐XIST. **P < .01

In further investigation, similar bioinformatics prediction inspired us that STAT3 might act as the target protein of XIST/miR‐146a‐5p axis (Figure 5A). Then, luciferase reporter assay demonstrated that miR‐146a‐5p mimics significantly combined with the STAT3 mRNA 3′‐UTR (Figure 5B). RT‐PCR assay demonstrated that STAT3 mRNA levels were up‐regulated in the LPS treatment, while decreased in the XIST knockdown (sh‐XIST) transfection (Figure 5C). Western blot analysis found that STAT3 protein was up‐regulated in the LPS treatment, while decreased in the XIST knockdown (sh‐XIST). Moreover, the co‐transfection of miR‐146a‐5p inhibitor with sh‐XIST increased the STAT3 protein (Figure 5D). In conclusion, these data suggested that STAT3 functioned as the downstream target of XIST/miR‐146a‐5p axis.

## 4 DISCUSSION

In the progression of ALI, inflammation response and apoptosis have been well documented in the development of acute lung injury (ALI).^14^ In advanced phase, the progression of ALI to a severe stage (oxygenation index <200) is known as acute respiratory distress syndrome (ARDS).^15^ Clinical epidemiological statistics find that ALI is associated with high morbidity and mortality rates worldwide. Given that the injury in pulmonary endothelial cells is a critical element and hallmark pathology underlying ALI/ARDS, the further research about endothelial cells injury is crucial for the aetiologic research and targeted research.

Here, in the LPS‐induced mice and HPMECs, lncRNA XIST was found to be up‐regulated as compared to the normal control. In multiple pathophysiological process, XIST had been reported to be involved in the occurrence of disease, including human cancers, diabetes mellitus and pneumonia. For instance, lncRNA XIST is robustly increased in the serum of patients with acute‐stage pneumonia and LPS‐induced human lung fibroblasts cells. Knockdown of XIST significantly reduced the LPS‐induced cell injury by up‐regulating cellular viability and decreasing apoptosis and inflammatory cytokines via JAK/STAT pathways.^16^ These data show that XIST may function as a vital regulator in the ALI.

**FIGURE 6:**
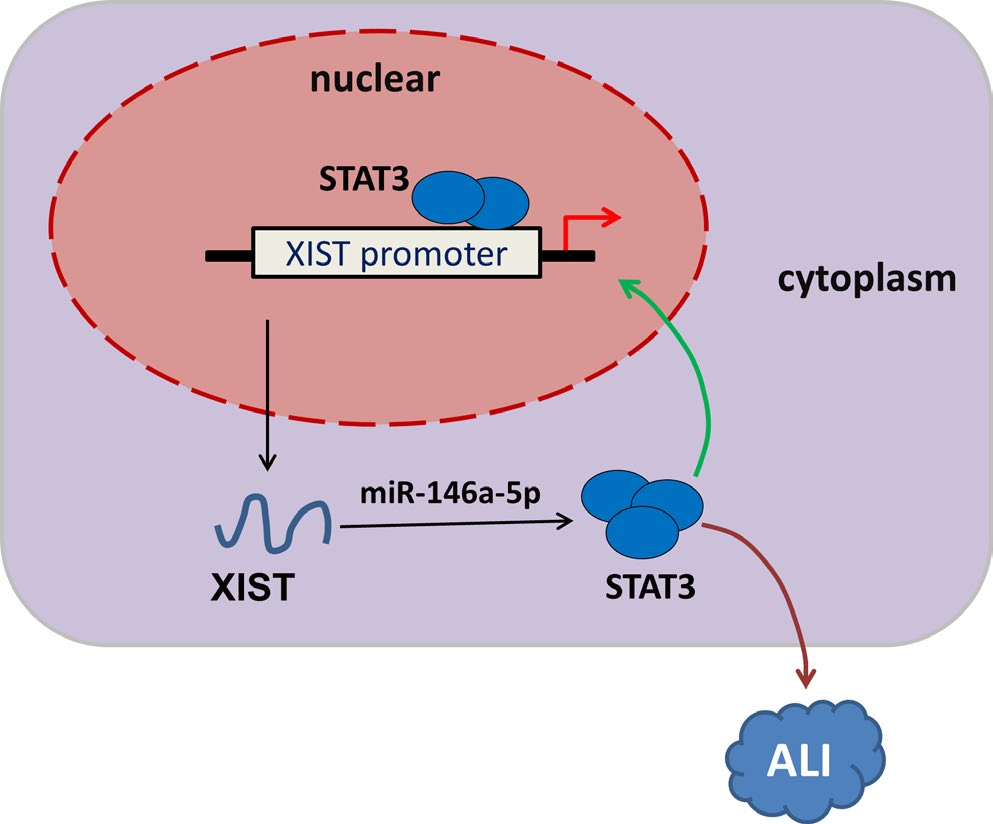
XIST/miR‐146a‐5p/STAT3 feedback loop accelerates the progression of LPS‐induced acute lung injury

Besides, STAT3, an important transcription factor in multiple pathophysiological processed, was not only the downstream target of XIST, but also the initiator for XIST. In the XIST promoter region, transcription factor STAT3 was found to bind with the potential domain. Increasing studies have demonstrated that STAT3 plays important roles in the apoptosis and inflammation of ALI.^17^, ^18^ Mechanistically, STAT3 accelerated the transcription abundance of XIST, forming a positive feedback loop of STAT3/XIST (Figure 6).

In functional experiments, we found that the LPS treatment could facilitate the apoptosis and inflammatory cytokine expression. Moreover, the knockdown of XIST could reduce the apoptosis and apoptotic rate in LPS‐induced pulmonary microvascular endothelial cells. The apoptosis of pulmonary endothelial cell may initiate or contribute the progression of series of lung diseases.^19^ Inflammation response is a remarkable marker for ALI, and the factors acting on endothelial cells may act as the potential therapeutic targets.^20^ In the present research, XIST was found to regulate the inflammation response and apoptosis of LPS‐induced pulmonary microvascular endothelial cells.

In the further research, we continue to investigate the mechanism of XIST regulating the pulmonary endothelial cell physical performance. The cytoplasmic distribution of XIST in LPS‐induced PMVECs led us to assume that XIST might function as miRNA sponge to regulate the target proteins. As respected, miR‐146a‐5p was predicted by the online bioinformatics tools and validated by the luciferase reporter assay. In the most molecular research examples, XIST exerts its regulation through sponging the downstream miRNAs. For example, lncRNA XIST and ZEB2, which were both targeted by miR‐367 and miR‐141, promote the TGF‐β‐induced epithelial‐mesenchymal transformation and metastasis of non‐small‐cell lung cancer.^21^ Similarly, XIST might regulate the ALI progression through regulating miR‐146a‐5p/STAT3 axis.

In conclusion, the present study demonstrated that XIST was dramatically up‐regulated in the LPS‐induced ALI models. Further findings showed that XIST functioned as a sponge of miR‐146a‐5p to mediate STAT3. Mechanistically, STAT3 accelerated the transcription abundance of XIST, forming a positive feedback loop of STAT3/XIST. Collectively, the results suggested that targeting the axis of XIST/miR‐146a‐5p/STAT3 may be effective in attenuating ALI.

## CONFLICT OF INTEREST

All authors declare no conflicts of interest.

## AUTHOR CONTRIBUTION

**Jun Li**: Data curation (equal); Supervision (equal). **Lei Xue**: Conceptualization (equal); Visualization (equal); Writing‐original draft (equal). **Yunfei Wu**: Data curation (equal); Software (equal). **Qiang Yang**: Formal analysis (equal); Software (equal). **Degang Liu**: Investigation (equal); Visualization (equal). **Changhui Yu**: Funding acquisition (equal); Supervision (equal). **Jiangzhou Peng**: Data curation (equal); Formal analysis (equal); Funding acquisition (equal); Investigation (equal); Supervision (equal); Validation (equal); Visualization (equal).

## Supporting information

Table S1

## Data Availability

No research data shared.
