# Supplementary material for: STAT3‐activated lncRNA XIST accelerates the inﬂammatory response and apoptosis of LPS‐induced acute lung injury
Source: J Cell Mol Med. 2021 Jun 11;25(14):6550–7. doi: 10.1111/jcmm.16653 (PMC8278113; doi:10.1111/jcmm.16653)
Supplement: Supplementary file 1 — Table S1 [file JCMM-25-6550-s001.docx]

**supplement Table S1**. Primers sequences for qRT-PCR and sequences of shRNA.

|  | Sequences |
| --- | --- |
| XIST | forward, 5’-CAGCAGCTTGACACACGGTA-3’  reverse, 5’-AAACACCAAAGTGGCATGTGA-3’ |
| miR-146a-5p | forward, 5’-GCAGCACATAATGGTTTGTG-3’  reverse, 5’-GCAGCACATCATGGTTTACA-3’ |
| STAT3 | forward, 5’-CTGTTGCTGCACGATCAGACA-3’  reverse, 5’-CTCAGTGGGGTTCATTACGGC-3’ |
| shRNA-XIST-1 | 5’-CAGCCUUAAGCGGAUUAUU-3’ |
| shRNA-XIST-2 | 5’-AACAGAGUAGAACCAUAUUU-3’ |
| shRNA-XIST-3 | 5’-GGUGGAGACCAAAAUUAGUUG-3’ |
| GAPDH | forward, 5’-GTCAACGGATTTGGTCTGTATT-3’  reverse, 5’-AGTCTTCTGGGTGGCAGTGAT-3’ |
